# Role of insulin/glucagon ratio and cell redox state in the hyperglycaemia induced by exposure to a 60-Hz magnetic field in rats

**DOI:** 10.1038/s41598-021-91228-w

**Published:** 2021-06-03

**Authors:** Gabriel Martiñón-Gutiérrez, María Luna-Castro, Rolando Hernández-Muñoz

**Affiliations:** Department of Cell Biology and Development, Institute of Cellular Physiology, Universidad Nacional Autónoma de México (UNAM), Ave. Universidad # 3000, Apdo. Postal 70-243, Coyoacán, 04510 Mexico City, Mexico

**Keywords:** Biochemistry, Biophysics, Cell biology, Endocrinology, Gastroenterology

## Abstract

The exposure to extremely low-frequency electromagnetic fields (EMFs) could adversely affect the endocrine system and cellular proliferative response. Nonetheless, the use of 60-Hz EMFs in the form of magneto-therapy exerts beneficial actions on human health but can also induce hyperglycaemia. Therefore, the present study was aimed to search for metabolic responses of fed or fasted male rats to a single EMF exposure. We performed a 15 min-single exposure to 60-Hz (3.8 mT, intensity) EMF, and determined serum levels of glucose, lipids, and indicators of cellular redox state and energy parameters. A single exposure to a 60-Hz EMF induced hyperglycaemia in both animal groups, and an attenuated second serum insulin peak. The 60-Hz EMF also decreased free fatty acids and lactate serum levels, oppositely increasing pyruvate and acetoacetate levels. Significant increases in blood glucose level and rat’s glucose metabolism were related to a more oxidized cellular redox state and variations in insulin and glucagon secretion. The 60-Hz EMF’s effects were not modified in animals previously subjected to chronic EMFs exposure (14 days). In conclusion, increased serum glucose levels and glucose metabolism induced by a single 60-Hz EMF exposure were closely related to the cellular redox state and the insulin/glucagon ratio.

## Introduction

Whenever electrical current flows, both electrical and magnetic fields are generated, which are known as electromagnetic fields (EMFs)^1^. These extremely low-frequency (ELF) EMFs have a long wavelength and range between 3 and 3000 Hz^2^, and its exposure could affect adversely human health, as epidemiological evidence exists indicating a correlation between exposure to ELF-EMF and childhood cancer incidence, Alzheimer disease, and even miscarriage. These alterations have been attributed to a possible association between DNA strand breaks and exposure to EMFs^3^. Although studies are showing the therapeutic effect of these EMFs, there is an evident lack of a comprehensive mechanism for explaining the discrepancy of the biological effect of EMFs on human health.

Indeed, positive effects of EMF therapy have been reported, in particular, in the rehabilitation of post-stroke patients and cancer treatment, mainly in combination with an anticancer drug^4,5^. From here, the use of EMFs, particularly that named “magneto-therapy, ” has had a notable increase in the last years, principally in rehabilitation treatment, where it provides a non-invasive and safe method to locally treat the site of injury, ameliorating pain, or to treat other systemic diseases like diabetes type 2^6,7^. Moreover, EMFs are seen as a prospective, non-invasive, and safe physical therapy strategy to accelerate bone repair by stimulating signalling cascades, which promotes osteogenesis and angiogenesis effectively^8^.

In this regard, EMFs have been found to reduce the release of pro-inflammatory cytokines such as tumour necrosis factor-α (TNF-α), interleukin (IL)-1β, and IL-6 in LPS-activated N9 microglial cells, suggesting that EMFs could represent a potential therapeutic approach in cerebral ischemic conditions^9^. In fact, pulsed EMFs exposure has become a potential therapeutic option, based on its angiogenesis-promoting properties^10^ and its ability to reduce infarct size and neuro-inflammation^11^, through decreasing pro-apoptotic mediators and increasing pro-survival molecules^12^, in response to activated signalling pathways induced by EMFs exposure^13^.

The exposure of cells to a 50-Hz magnetic field could induce EGF receptor clustering and phosphorylation; EMFs can accelerate neural differentiation of bone marrow-derived macrophages via EGF receptor activation^14^. Therefore, changes in EGFR status are also accompanied by changes in the level of reactive oxygen species (ROS) after EMFs exposure^15,16^, increasing enzymatic antioxidant activity and improving functional and mental activity^17^.

On the other hand, exposure to EMFs could participate in the response to stress by affecting cell metabolism especially that linked to metabolic energy production. EMFs suppress lactate anabolism, probably dependent on the adrenergic status of the animal. Application of EMFs to hind-limb ischemic rats does not significantly increase blood levels of lactate and glucose, but decreases levels of free fatty acids (FFA)^18^. However, in normal animals subjected to stress, 50-Hz EMFs exposure tends to decrease plasma ACTH and glucose, and significantly decreases plasma lactate levels in stressed rats^19^. In addition, these EMFs decrease plasma levels of total cholesterol and phospholipids, and downregulate liver diacylglycerol acyltransferase-2 mRNA expression^20^. Although the mechanisms for the discrepant effects of EMFs on glucose and lipid metabolism are not well understood yet, effects on insulin secretion could be involved.

In 1991, it was reported that, in rats chronically exposed to uniform constant MFs, blood glucose increased slightly, insulin release decreased, and glucagon content increased, implicating probably a temporarily diabetic-like response in the treated-rats^21^. These effects were also confirmed by exposing HITT15 cells to EMFs at 5 mT, which decreased glucose-stimulated insulin secretion, ATP/ADP ratio, membrane depolarization, and cytosolic free calcium ion concentration^22^. Therefore, the serum glucose levels and the glucose-stimulation of insulin secretion could depend on the cellular metabolic conditions and EMFs treatment protocol.

The present work is an attempt to gain some insight on the metabolic responses of fed or fasted male rats to a single exposure of extremely-low frequency EMFs, measuring serum levels of glucose, lipids, and indicators of cellular redox state and energy parameters, as well as to perform a metabolic tracing of glucose in the whole animal. Our data indicate that EMFs induced a hyperglycaemic state in both fed and fasted rats, accompanied by a drastic attenuation of a second serum insulin peak. Indeed, increased serum glucose levels were closely related with the cellular redox state and the insulin/glucagon ratio.

## Results

### Effects of the single exposure to EMFs on glucose, lipids, and redox pair-metabolites in fed and fasted rats

**Figure 1:**
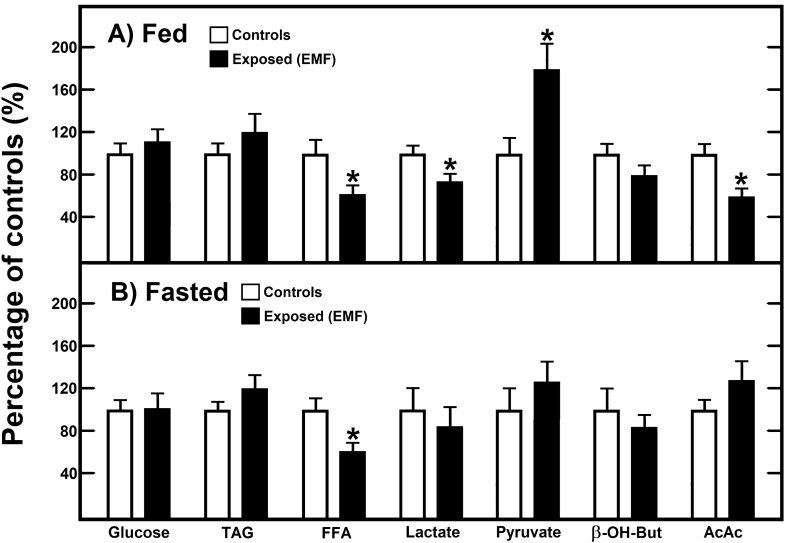
Effects of the single exposure to EMFs on glucose, lipids, and redox pair-metabolites in fed and fasted rats. Results are the mean ± SE of three individual observations per experimental group (6 control animals, and 6 treated rats). Blood samples were immediately taken after 15 min exposure to EMFs. Experimental groups are designated by symbols at the upper part of each panel. In panel A, control values (100%) for fed animals were glucose = 101 ± 9 mg·dL^−1^, triacylglycerols (TAG) = 174 ± 15 mg·dL^−1^, free fatty acids (FFA) = 3.8 ± 0.5 µmol·dL^−1^, lactate = 1.26 ± 0.08 mmol·L^−1^, pyruvate = 0.06 ± 0.01 mmol·L^−1^, β-hydroxybutyrate (β-OH-but) = 0.16 ± 0.02 mmol·L^−1^, and acetoacetate (AcAc) = 0.32 ± 0.06 mmol·L^−1^. In panel B control values for fasted animals were: glucose = 77 ± 5 mg·dL^−1^, TAG = 167 ± 14 mg·dL^−1^, FFA = 5.2 ± 0.5 µmol·dL^−1^, lactate = 1.89 ± 0.37 mmol·L^−1^, pyruvate = 0.08 ± 0.02 mmol·L^−1^, β-OH-but = 0.58 ± 0.12 mmol·L^−1^, and AcAc = 0.18 ± 0.02 mmol·L^−1^. Statistical significance: *p < 0.01 versus control group.

Animals were subjected to a single 15 min-exposure of 60 Hz-EMFs (time zero), and euthanized immediately after obtaining blood samples. In fed animals, the EMFs did not affect significantly serum levels of glucose or triacylglycerols (TAG), but decreased largely FFA serum levels (Fig. 1A). Moreover, 60 Hz-EMFs exerted an opposite effect on the redox-pair metabolites: lactate was decreased and pyruvate was drastically increased in the serum; in addition, serum levels of acetoacetate (AcAc) were also significantly decreased (Fig. 1A).

In overnight fasted animals, in which the metabolic environment was changed, i.e., decreased serum level of glucose, increased FFA, and lactate and ketone bodies, the exposure to 60-Hz EMFs had not significant effects on these parameters, except that 60-Hz EMFs also decreased levels of FFA (Fig. 1B). Thus, depending on the metabolic scenario, a single exposure to 60-Hz EMFs exerts significant metabolic changes in rats.

### Time-course of 60-Hz EMFs’ effects on glucose, lactate, and pyruvate in fed and fasted rats

**Figure 2:**
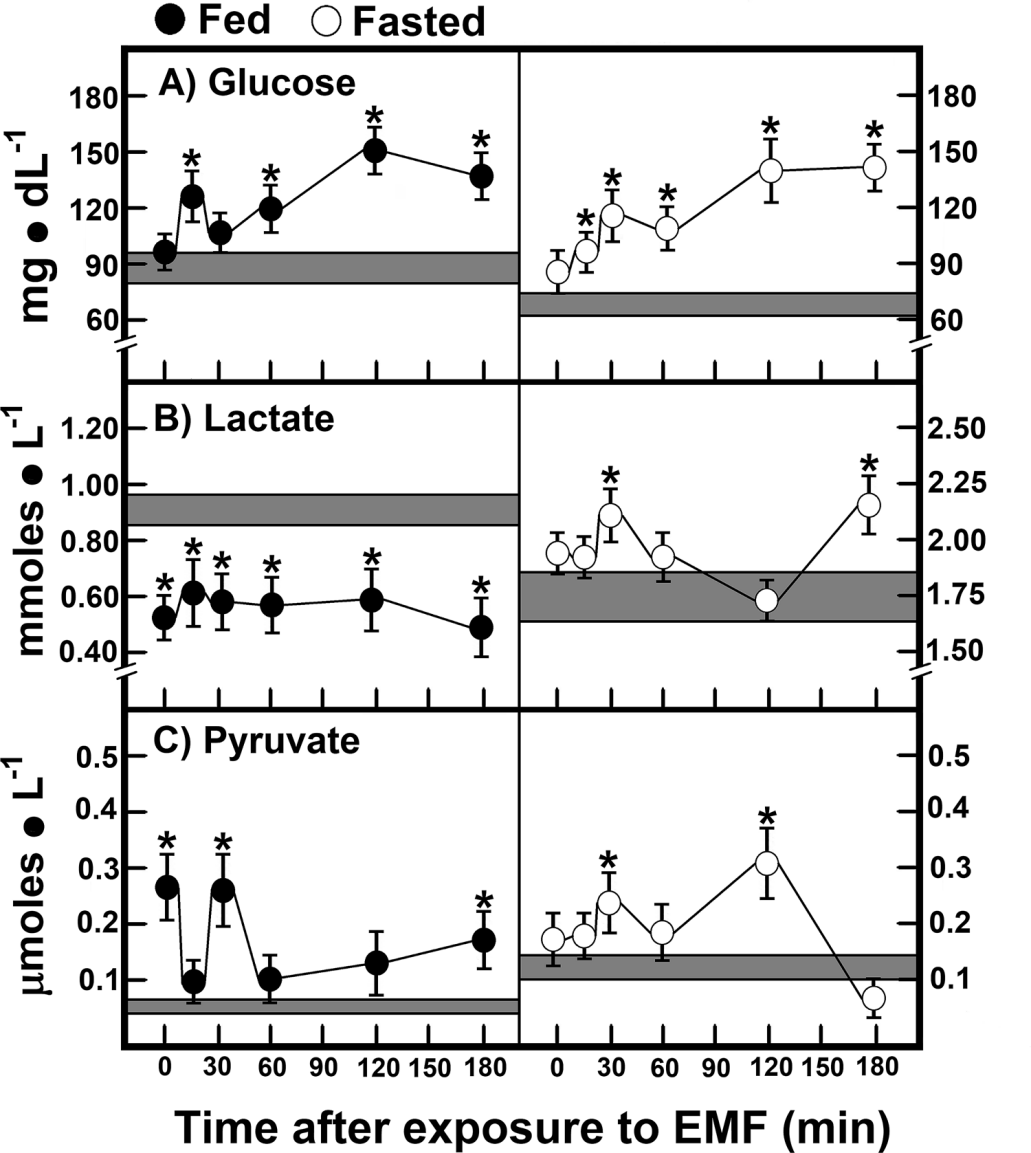
Time-course of EMFs’ effects on glucose, lactate, and pyruvate in fed and fasted rats. Results are the mean ± SE of three individual observations per experimental group and point (6 control animals vs. 6 treated rats), for glucose (panels A), lactate (panels B), and pyruvate (panels C). Experimental groups are designated by symbols at the upper part of the figure. Statistical significance: *p < 0.01 versus control group.

Since we did not find higher levels of glucose in animals subjected to a 15-min exposure to 60-Hz EMFs, as previously reported^21^, we looked for a time-course of 60-Hz EMFs’ effects on some parameters. In fed animals, serum levels of glucose increased at 15 min after exposure, returned to the control levels (30 min), and started again to increase 60 min after exposure (Fig. 2A). In these animals, lactate was decreased early, and this significant diminution was maintained (3 h, Fig. 2B). On the contrary, 60-Hz EMFs promoted three peaks of augmented serum levels of pyruvate at 0, 30, and 180 min (Fig. 2C), where the lactate/pyruvate ratio was largely decreased (not shown).

Interestingly, in animals with an overnight fast, a hyperglycaemic status was observed after exposure to 60-Hz EMFs, reaching the same higher blood levels for glucose as in fed animals at the end of the experiment (Fig. 2A). However, lactate levels (which were much higher in these animals) did not decrease, but rather showed two increasing peaks at 30 and 180 min after treatment (Fig. 2B). The 60-Hz EMFs exposure promoted two increments of serum pyruvate at 30 and 120 min (Fig. 2C). Therefore, increases of serum glucose seem to coincide with lower lactate/pyruvate ratios (more oxidized state) after 60-Hz EMFs treatment (Fig. 2).

### Curve of tolerance to glucose and levels of insulin and glucagon in rats treated with EMFs

**Figure 3:**
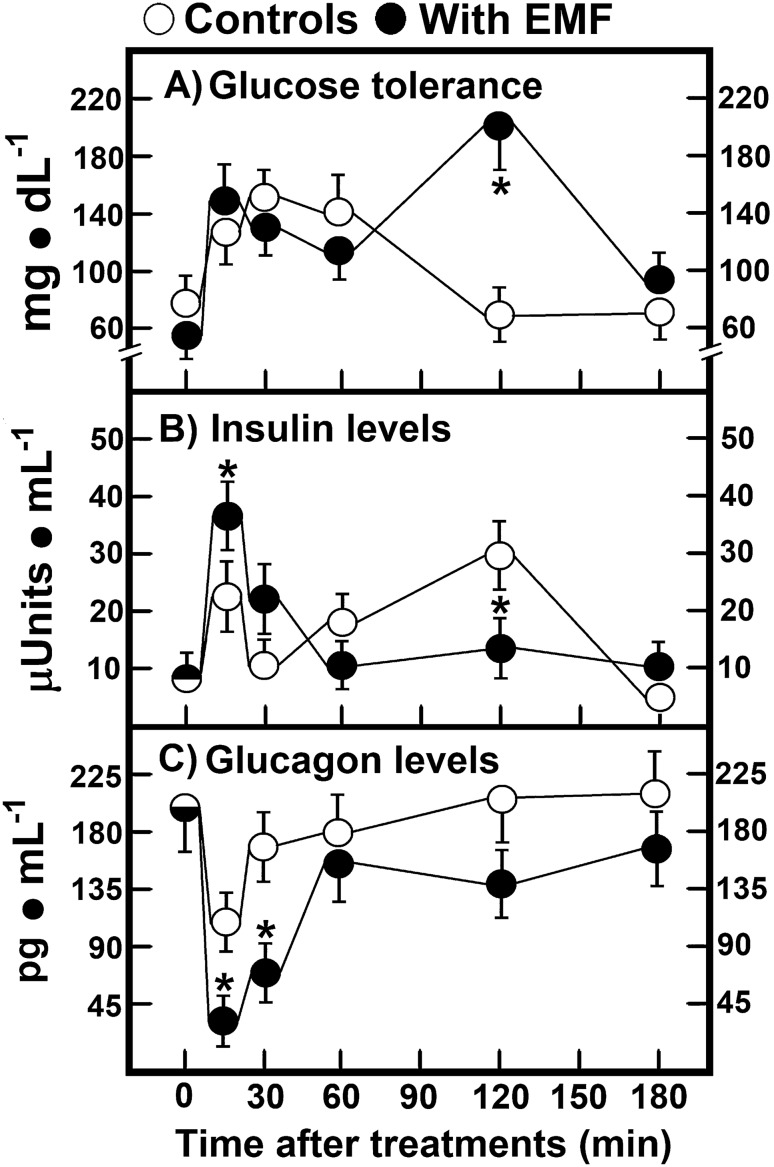
Curve of glucose tolerance and levels of insulin and glucagon in rats treated with EMFs. Results are the mean ± SE of five individual observations per experimental group and point (5 control animals vs. 5 treated rats) for glucose (panel A), insulin (panel B), and glucagon (panel C). Experimental groups are designated by symbols at the upper part of the figure. Statistical significance as indicated in Fig. 1.

Another set of rats were fasted overnight, administered with a glucose load of 2 g·kg^−1^, and subjected to a 15 min-exposure of 60-Hz EMFs. Control (sham) animals depicted a “typical” absorption and further decay of blood glucose levels assumed to be due to its utilization. In rats treated with EMFs, we noted that, during the first 90 min, glucose tolerance was similar to that of the control group, but at 120 min after glucose administration, a robust hyperglycaemia occurred, which could be considered a “diabetic-like response” curve (Fig. 3A). In this context, control animals elicited two peaks of insulin secretion, the first at 15 min and the second 2 h after treatment. Although EMFs indeed increased the first peak of insulin when compared with control animals, the second insulin peak was practically abolished by the treatment with 60-Hz EMFs (Fig. 3B). Interestingly, in control and treated animals, serum levels of glucagon showed an opposite pattern of release when compared to that of insulin. However, during the first 30 min of the tolerance curve, animals treated with 60-Hz EMFs had the lowest serum levels of this hormone (Fig. 3C). Therefore, the insulin/glucagon ratio could be involved in the hyperglycaemic effect of 60-Hz EMFs in these rats.

### In vivo oxidation of U-(^14^C)-glucose and its incorporation into glycogen and glycerol-containing lipids in rats treated with EMFs

**Figure 4:**
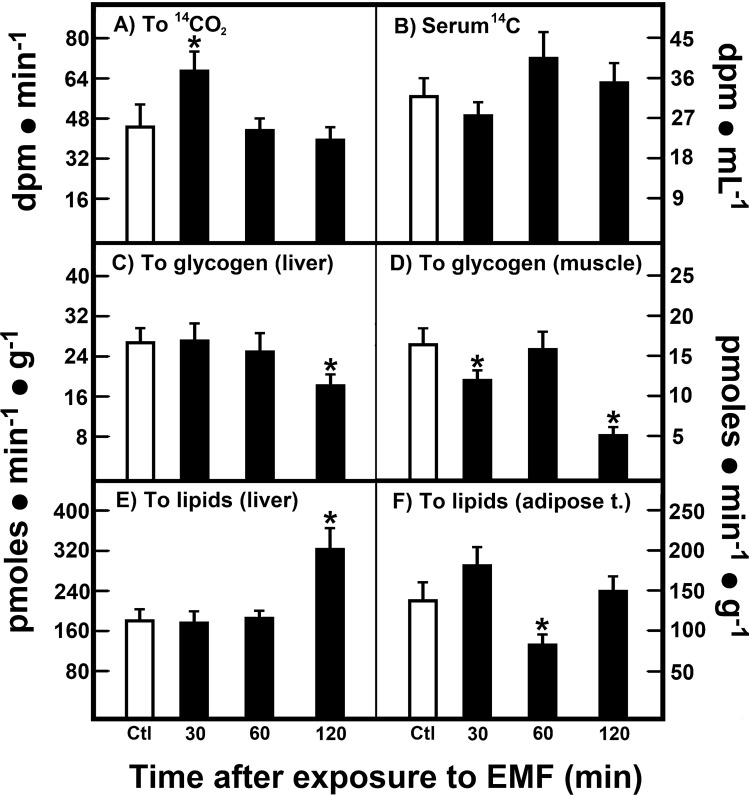
In vivo oxidation of U-(^14^C)-glucose and its incorporation into glycogen and glycerol-containing lipids in rats treated with EMFs. Results are the mean ± SE of four individual observations per experimental group and point (8 control animals and 12 treated rats), for glucose oxidation (panel A), blood glucose (panel B), its incorporation into glycogen in the liver (panel C) and in the muscle (panel D). In addition, incorporation of labelled glycerol into TAG is also depicted in the liver (panel E), as well as in the adipose tissue (panel F). Statistical significance as indicated in Fig. 1.

Another set of fed rats received 0.5 mg of glucose-containing 4 µCi (148 mBq) of (U-^14^C)-glucose, and was subjected to a 15 min-exposure of 60-Hz EMFs. Animals exposed to 60-Hz EMFs showed an increased glucose oxidation, as reflected by the production of ^14^CO_2_ (Fig. 4A), which was accompanied by a tendency of decreasing blood level of ^14^C-glucose (Fig. 4B) 30 min after 60-Hz EMFs treatment. Synthesis of liver glycogen, as assessed by incorporation of glucose into glycogen, was decreased at 120 min after exposure (Fig. 4C), coinciding with the abatement of the second peak of insulin secretion (Fig. 3B). As to muscular glycogen, treatment with 60-Hz EMFs induced two decrements in its synthesis, at 30 and 120 min (Fig. 4D); these effects also seemed to be related with the initial serum insulin (Fig. 3B), as well as with the augmented glucose oxidation (Fig. 4A). Moreover, whereas 60-Hz EMFs promoted lipogenesis (as assessed by FFA esterification to ^14^C-glycerol) in the liver (120 min; Fig. 4E), in the epididymal adipose tissue, a transient decrease occurred in the rate of lipogenesis 60 min after 60-Hz EMFs exposure (Fig. 4F).

### Correlations among serum glucose levels, insulin, glucagon, and redox-pair metabolites

**Figure 5:**
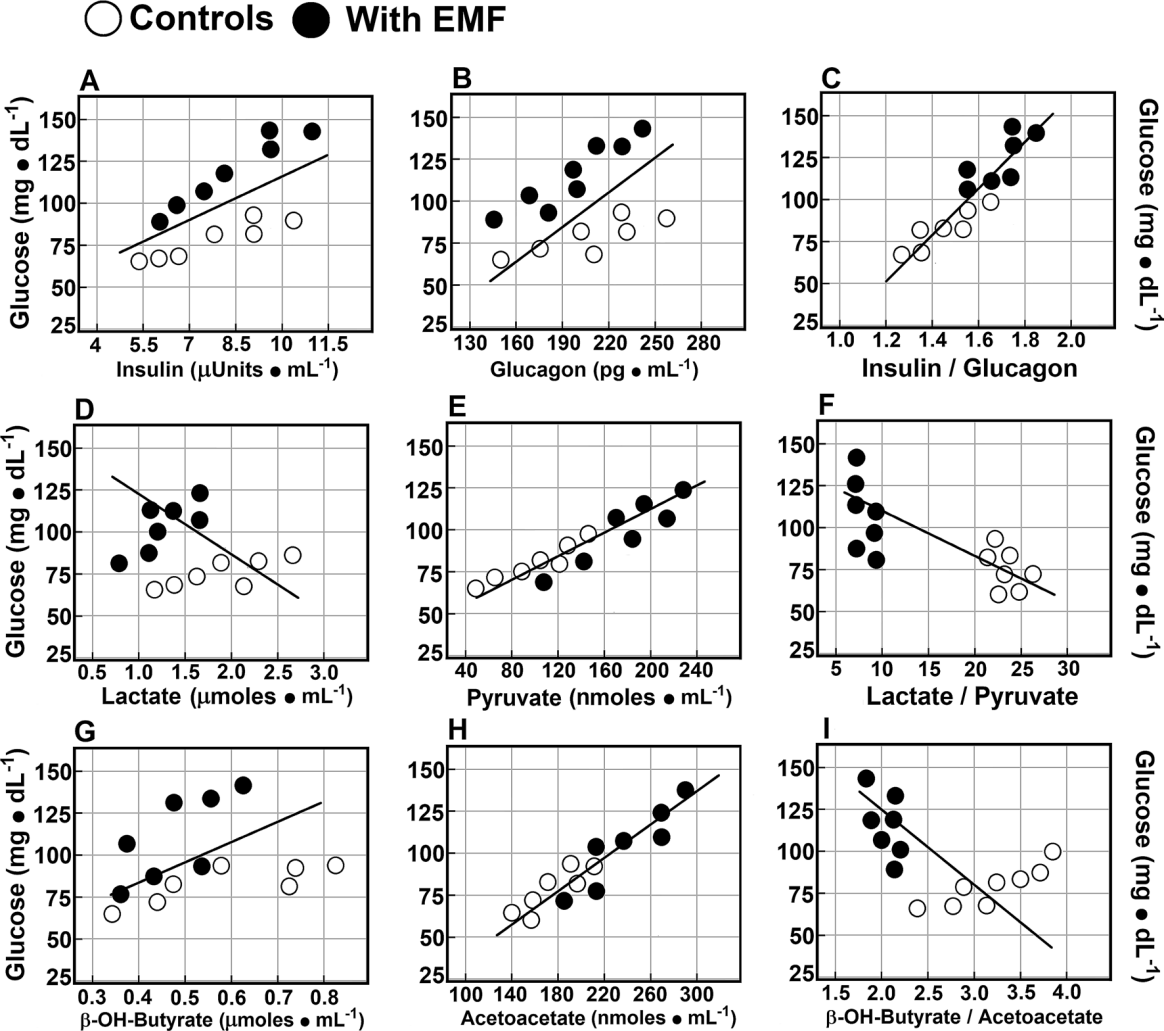
Correlations among serum glucose levels, insulin, glucagon, and redox-pair metabolites. Scatter plots show the relation between two sets of data. This relation is summarized in the Pearson’s correlation coefficient (r) for each relation in each scatter plot: panel A, glucose vs. insulin levels (r = 0.687, *p* < 0.01), panel B: glucose vs. glucagon levels (r = 0.403, *p* < 0.05), and panel C, glucose against the insulin/glucagon ratio (r = 0.965, *p* < 0.001). Panel D shows correlations of glucose vs. lactate levels (r =  − 0.132, n.s.), panel E, glucose against pyruvate levels (r = 0.993, *p* < 0.001), and panel F, glucose levels vs. the lactate/pyruvate ratio (r =  − 0.806, *p* < 0.001). Finally, glucose against β-OH-but levels (r = 0.146, n.s) in panel G, panel H, glucose vs. AcAc levels (r =  − 0.963, *p* < 0.001), and panel I, glucose levels against the β-OH-but/AcAc ratio (r =  − 0.584, *p* < 0.01).

We looked for possible relationships between the hyperglycaemic status induced by 60-Hz EMFs exposure and the serum levels of pancreatic hormones, as well as with redox-pair metabolites (Fig. 5). The levels of glucose in control and treated rats correlated directly with fluctuations in insulin levels (r = 0.687, *p* < 0.01; Fig. 5A), with those of glucagon (r = 0.403, *p* < 0.05; Fig. 5B), and much better with the insulin/glucagon ratio (r = 0.965, *p* < 0.001; Fig. 5C). Regarding lactate and pyruvate, serum glucose did not correlate with lactate (r =  − 0.132, n.s.; Fig. 5D), but a very high significant relationship was found with pyruvate (r = 0.993, *p* < 0.001; Fig. 5E); glucose level also showed an inverse correlation with the lactate/pyruvate ratio (r =  − 0.806, *p* < 0.001; Fig. 5F), indicating the relevance of fluctuations in pyruvate levels. In the same context, glucose did not correlate significantly with β-hydroxy-butyrate (β-OH-but; r = 0.146, n.s.; Fig. 5G), but correlated highly and directly with levels for AcAc (r =  − 0.963, *p* < 0.001; Fig. 5H). Moreover, glucose also showed an inverse correlation with the β-OH-but /AcAc ratio (r =  − 0.584, *p* < 0.01; Fig. 5I); thus, glucose increase was highly correlated with an enhancement in oxidized metabolic products, namely pyruvate and AcAc (Fig. 5).

### Effects of chronic exposure to EMFs on rat blood glucose, lipids, and parameters indicative of redox state and energy status

Most of the reported effects of EMFs on cell proliferation, cell signalling, oxidant status, or metabolic changes have been obtained through chronic exposure to EMFs in animals or isolated cells^2^. Therefore, we searched whether the acute response to EMFs remains even after exposing the animals for 14 consecutive days to 60- Hz MFs. For this, a set of rats was exposed for 15 min to 60 Hz-MFs per day for two weeks, as indicated in the Methods section. Table 1 shows that this chronically-treated group also elicited a significant hyperglycaemia, but the decrease in FFA serum levels was not seen, which could be related with a more oxidized mitochondrial NAD/NADH redox state, induced by the 60-HzMFs. The lactate/pyruvate ratio was decreased and, consequently, the NAD/NADH ratio was augmented in both groups, but through a different mechanism; in the group of a single exposure, pyruvate levels were increased, whereas in the chronic group, the levels of lactate were diminished (Table 1). Total ketone bodies level was not significantly changed in both groups, but, in the chronic group, the β-OH-but/AcAc ratio was decreased, indicating a more oxidized NAD/NADH status, presumably reflecting liver mitochondria redox state (Table 1). Finally, when examining blood energy parameters (ATP/ADP ratio, energy charge, and total adenine nucleotides), we did not find any significant difference in these indicators in either the “acute” or the “chronic” groups (Table 1). Therefore, chronic application of 60-Hz EMFs does not induce refractory actions on the effects of a single 60-Hz EMFs exposure in rats.

**Table 1:** Effects of chronic exposure to EMFs on rat blood glucose, lipids, and parameters indicative of redox state and energy status.

Parameter	Fasted (single)		Fasted (chronic)	
	Controls	With EMF	Control	With EMF
Glucose (mg·dL^−1^)	77 ± 5	119 ± 15*	72 ± 7	110 ± 5*
TAG (mg·dL^−1^)	167 ± 14	203 ± 26	187 ± 19	162 ± 20
FFA (µmoles·dL^−1^)	5.2 ± 0.5	3.2 ± 0.3*	6.5 ± 0.8	6.1 ± 0.6
Lactate (mM)	1.89 ± 0.37	2.01 ± 0.30	1.61 ± 0.25	0.89 ± 0.14*
Pyruvate (mM)	0.08 ± 0.02	0.19 ± 0.03*	0.07 ± 0.02	0.13 ± 0.03
Lactate/Pyruvate	23.6 ± 4.9	10.6 ± 1.8*	22.5 ± 4.0	6.7 ± 1.6*
NAD + /NADH (Cyto)	382 ± 79	852 ± 136*	392 ± 60	1316 ± 261*
β-OH-butyrate (mM)	0.58 ± 0.12	0.49 ± 0.06	0.33 ± 0.07	0.27 ± 0.06
Acetoacetate (mM)	0.18 ± 0.02	0.24 ± 0.03	0.14 ± 0.02	0.23 ± 0.03
β-OH-but/AcAc	3.2 ± 0.5	2.1 ± 0.3	2.4 ± 0.4	1.2 ± 0.2*
NAD + /NADH (Mito)	7.6 ± 1.1	11.5 ± 1.7	10.1 ± 1.7	19.6 ± 3.1*
ATP (mM)	0.77 ± 0.05	0.72 ± 0.04	0.71 ± 0.05	0.58 ± 0.05
ADP (mM)	0.10 ± 0.01	0.09 ± 0.01	0.17 ± 0.02	0.16 ± 0.03
AMP (mM)	0.06 ± 0.01	0.04 ± 0.01	0.09 ± 0.01	0.08 ± 0.02
ATP/ADP	7.7 ± 0.8	8.0 ± 0.6	4.2 ± 0.5	3.6 ± 0.7
Total nucleotides	0.93 ± 0.12	0.85 ± 0.11	0.97 ± 0.14	0.82 ± 0.11
Energy charge	0.88 ± 0.03	0.89 ± 0.04	0.81 ± 0.04	0.80 ± 0.03

Results are the mean ± SE of four individual observations per experimental group, which consisted of rats exposed to 60-Hz EMFs during 15 min/day for 14 consecutive days before the single (acute) exposure at the day of the experiment (8 control animals vs. 8 treated rats), and blood samples were taken 30 min after ending exposure. Abbreviations: β-OH-but, β-hydroxybutyrate, AcAc, acetoacetate, Cyto, cytoplasm, and Mito, mitochondria. Statistical significance: *p < 0.01 versus control group.

## Discussion

Interaction between biological systems and external MFs has received considerable attention in the last years, but there are discrepant results that complicate reaching a consensus on this interaction. We must take into account the complexity of biological systems, as well as the different protocols in applying a variety of schemes for EMFs treatment^23^. In our experimental conditions, in normal fed or fasted male Wistar rats, we found that EMFs induced a hyperglycaemic state in both fed and fasted rats, accompanied by a drastic decrease in serum FFA levels; these effects also coincided with an attenuation of a second serum insulin peak. Indeed, increased serum glucose levels were closely related with the cellular redox state, mainly favoured by a more oxidized state and the insulin/glucagon ratio.

An increased, albeit slight, blood glucose level accompanied by a diminished insulin content has already been reported in rats chronically exposed to uniform constant EMFs^21^. This effect was related to an adrenergic effect induced by EMFs, where adrenaline would favour the stimulation of glucagon secretion over that of insulin at the level of the pancreatic Langerhans islets^24,25^. In this regard, there are clear discrepancies about the effects of EMFs on insulin secretion in rats, which seem to depend on the applied frequency and magnitude of the magnetic intensity flux^26,27^. From here, the in vitro experiments, where EMFs exposure increases cell number under apoptotic culture conditions of pancreatic cells, could lead to new therapeutic concepts in the treatment of diabetes^22^.

The extremely low-frequency EMFs can affect Ca^2+^ mobilization and signalling, which can be involved in insulin release, and it has been found that 60-Hz EMFs stimulation increases insulin secretion in diabetic and normal rats^28,29^. Indeed, it has been shown that EMFs with the same frequency of the natural frequencies of β-cell increase insulin secretion, which may indicate strong energy (ATP) coupling between insulin secretion and EMFs^23^. In contrast, EMFs can decrease glucose-stimulated insulin secretion and the ATP/ADP ratio in isolated cells, these effects are accompanied by a membrane depolarization and an increased cytosolic free calcium ion concentration^22^. In other conditions, increased levels of glucose were accompanied by significant variations in insulin secretion but without significant changes in glucagon levels after EMFs exposure (Fig. 2), which indicates that the insulin/glucagon ratio exerts a better control on glycaemia (Fig. 5), and appears as a main factor in the EMFs-mediated effect on blood glucose. The control of cholesterol by insulin and glucagon is regulated by plasma amino acids, being the insulin/glucagon ratio an early metabolic index controlling these effects^30^. In addition, an increased insulin/glucagon ratio after reperfusion correlates with the arterial ketone body ratio during recovery of graft liver function after in vivo liver transplantation^31^.

Besides, chronic EMFs exposure is able to decrease the high levels of FFA and TG found in rats subjected to ischemia, probably affecting FFA and TAG metabolism by enhancing availability of metabolic energy^18^. Moreover, the same research group reported previously that EMFs tend to suppress the stress-induced increase in plasma ACTH and glucose levels, reducing plasma lactate level, without affecting pyruvate^19^. In the present study, we found that 60-Hz EMFs decreased FFA levels without affecting those of TAG, even in a stressed state, like an overnight fast (Fig. 1); in addition, 60-Hz EMFs diminished lactate levels in fed animals whereas augmenting pyruvate (Fig. 2). On the contrary, in fasted rats, lactate was not decreased but pyruvate was significantly increased (Fig. 2); as a result, in both metabolic situations, the lactate/pyruvate ratio was diminished and, therefore, the NAD/NADH redox potential was significantly increased (Table 1). Besides, the phosphorylation potential and the mitochondrial functionality to carry out oxidative phosphorylation, the cell capacity to oxidize substrates would depend upon another thermodynamic factor, known as the oxidation–reduction (redox) potential^32^. From here, cell redox state is recognized as an important regulatory factor of many metabolic fluxes^33^.

We have shown that blood variations in the redox-pair metabolites, such as lactate and pyruvate, and mainly those in ketone bodies (β-OH-but and AcAc), largely reflect those occurring in the liver^34^. Here, the effects of 60-Hz EMFs clearly favoured a more oxidized cellular redox state, which could explain the in vivo changes in glucose metabolism in rats exposed to 60-Hz EMFs (Fig. 4). For instance, these animals showed an increased glucose oxidation, which we think was due to an insulin-dependent effect in the muscles, which was accompanied by decrements in glycogen synthesis (Fig. 4D), but was not seen in the liver coinciding with the abatement of the second insulin peak, and contrasting with the enhanced liver glycogen accumulation found after chronic 60-Hz EMFs exposure^35^. In the same context, we recorded a transient decrease in the rate of lipogenesis in the epididymal adipose tissue, which is also a metabolic pathway dependent on insulin action (Fig. 4F). Interestingly, 60-Hz EMFs promoted lipogenesis later in the liver (Fig. 4E), which was not seen due to its effects on TAG mobilization from the liver. Our data agree with previous results suggesting that putative effects on serum lipids are more significant in longer exposure times, different frequencies, intensities, and durations of 60-Hz EMFs exposure^36^. Indeed, assuming that our local geomagnetic field’s size and amplitude ranged from 25 to 65 μT, we are using a magnetic flux density 50 to 85 times above this range, and magnetic flux densities elicit differential biological responses. For instance, the 1 and 100 μT densities of 50-Hz EMFs had more immunological effects than EMFs with higher densities; therefore, activating anti-inflam matory effects in rats, by down-modulating pro-inflammatory cytokines and inducing the anti-inflammatory cytokine IL-10^37^. In the same context, the expression levels of c-Maf, STAT6, and RORα in the thymus were not significantly changed at different magnetic flux densities, whereas expression of RORα and c-Maf were significantly downregulated at 1 and 100 μT densities in the spleen, thus showing differential effects according to the magnetic flux density, as well as organ-specific^38^.

As mentioned above, another important factor involved in the metabolic effects induced by EMFs exposure can be the production of cell energetic parameters (ATP availability). Plasma glucose levels respond to EMFs in the kilohertz range elevating insulin secretion, where EMFs would reach the internal structures and increase ATP synthesis^39,40^. Indeed, EMFs exposure attenuated KCl-stimulated insulin secretion influencing the increase in ATP/ADP and membrane depolarization, and attenuating the increase of [Ca^2+^]i that is linked to a depolarization in isolated β-cells^41,42^. In our study, neither with a single (acute) nor with a chronic exposure to 60-Hz EMFs, did we detect any significant change in the energy parameters in the blood from these animals (Table 1), which would suggest that metabolic energy availability was not an important factor in the changes in glucose metabolism and insulin secretion elicited by 60-Hz EMFs under our experimental conditions. However, we have also demonstrated that changes in blood energetic parameters are very closely related with those in the liver^43^; therefore, we do not know whether our protocol for the 60-Hz EMFs treatment actually affected pancreatic energy parameters in our experimental animals, similarly to what happens in isolated β-cells. This and other related issues are objectives for future research. Therefore, despite that we can assume that metabolic energy in the form of ATP and its effects on calcium mobilization are involved in the EMFs’ effects on insulin secretion, we cannot rule out the possibility that a stimulated symphatetic nerve excitation, induced by exposure to EMFs, would change hormonal release.

In conclusion, our data indicate that 60-Hz EMFs induced a hyperglycaemic state in both fed and fasted rats, accompanied by a severe attenuation of a second serum insulin peak. Indeed, increased serum glucose levels were closely related to the cellular redox state and the insulin/glucagon ratio; moreover, glucose metabolism in the whole animal also coincided with variations in insulin and glucagon secretion, probably, dependent on the cell redox state. Most of these effects were also recorded in animals subjected to a chronic EMFs exposure, indicating that there were no refractory actions from a continuous application of electromagnetic therapy. Hence, since the blood levels of insulin and those of glucagon can be largely influenced by the cell redox state, as a possible new revealed factor controlling hormone release, the dietary or pharmacological management of blood redox state could have a clinical impact in the treatment of hyperglycaemic situations.

## Material and methods

### Materials

Enzymes and coenzymes for enzyme-coupled reactions for metabolite determinations, as well as other analytical reagents, were purchased from Sigma-Aldrich Chemical Co. (St. Louis, MO, USA). The U-^14^C-glucose (sp. act. 9.9 GBq/mol; NEN Radiochemicals, Boston, MA, USA).

### Animal subjects and ethics

Our research was approved by the Animal Experiments Institutional Ethics Committee of the Instituto de Fisiología Celular, UNAM, according to the Federal Regulations for Animal Experimentation (Ministry of Agriculture, SAGARPA, Mexico), and in compliance with the ARRIVE guidelines. Seventy male 10-week-old Wistar rats (240–270 g of b.w.) were obtained from the certified bioterium of the Instituto de Fisiología Celular, UNAM; animals were housed under a 12:12 h light/dark cycle with free access to food (diet chow 500I, LabDiet Co, St Louis, MO, USA) and water.

### Animal treatment (exposure to MFs)

The EMF exposure system is composed of a high voltage transformer, a constant voltage unit, and EF exposure cages, which was designed for a rat or a smaller animal, as a cylindrical plastic cage (12 inches-diameter and height). This cage has two stainless steel electrodes, connected to a plastic wrapped solenoid that laterally surrounds the cylindrical cage. We modulated the intensity of LF-EMF using the transformer. Each coil in the solenoid had 200 turns of an soft copper wire, which was connected in series to 120-V AC power, producing a 60-Hz electromagnetic field set to 3.8 mT root mean square (rms) amplitude using a Gauss meter (Model 410; Lake Shore Cryotronics, Westerville, OH, USA) in the vertical direction at the centre of the system^44^; the temperature was maintained steady at the inside of the cylindrical chamber by an exterior fan. The experimental group was subjected to a single exposure at a magnetic intensity of 3.8 mT, 60-Hz, extreme low frequency EMF, for 15 min in the cylindrical cage. Sham control animals were placed in the same cage with the coils turned off, being only exposed to the local ambient geomagnetic field; the 60-Hz-EMFs exposure was always done between 10:00 and 11:30 h. At indicated post-treatment (60- Hz-EMFs exposure) times, blood samples were collected through cardiac puncture and animals were euthanized with an overdose of sodium pentobarbital. Another set of rats was fasted overnight before treatment and euthanasia. We also evaluated the effects of a chronic exposure to 60-Hz EMFs on the metabolic parameters. For this, a set of rats was exposed for 15 min to 60-Hz EMFs per day for two weeks; then, animals were fasted overnight on the 15^th^ day, and then subjected to a single (acute) 60-Hz EMFs’ exposure.

### Sera sampling and clinical tests

Serum was obtained by centrifuging uncoagulated whole blood; in these sera**,** glucose and TAG were measured through standardized procedures with kits from SPINREACT S.A. (Spain). Serum levels of total fatty acids (FFA) were determined by pH titration, according to Novak^45^. Serum insulin and glucagon levels were measured with kits from RayBiotech (Peachtree Corners, GA, USA).

### Curve of glucose tolerance

After an overnight fast, the animals were orally given 2 g·kg^−1^ of glucose and exposed 15 min to the generated 60-Hz EMFs. Blood samples were taken from a tail vein at 15, 30, 60, 120, and 180 min after glucose administration (n = 4, per experimental point), placed in capillary tubes and centrifuged to obtain sera. Thereafter, we performed the determinations of glucose, insulin, and glucagon in these serum samples.

### Analytical procedures

In neutralised with 4 mol·L^−1^ K_2_CO_3_ serum samples from perchloric acid-extracts, the redox-pair metabolites: lactate, pyruvate, β-OH-but, and AcAc were enzymatically determined, as described before^34^. For adenine nucleotides (ATP, ADP, and AMP) determination, whole blood was directly poured for extraction into ice-cold 8% perchloric acid; after centrifugation, the sample was neutralised with 4 mol·L^−1^ K_2_CO_3_. Adenine nucleotides in the neutralised acid-extract were quantified by reversed-phase high performance liquid chromatography (HPLC)^46^.

### In vivo quantification of (^14^C)-glucose oxidation and its incorporation to (^14^C)- glycogen, as well as of (^14^C)-glycerol into lipids

A set of rats was intraperitoneally injected with 0.2 mL of saline-dissolved 0.5 mg of glucose-containing 4 µCi (148 mBq) of (U-^14^C)-glucose. After administration, animals were individually placed in the metabolic cage, especially designed for rats, and the experimental group was now exposed to 60-Hz EMFs for 15 min (time zero). Afterwards, animals were euthanized at 15, 60, and 120 min, and samples were taken from serum, liver, caudofemoralis muscle, and the epididymal white adipose tissue. The ^14^CO_2_ was trapped using a strong base, hyamine hydrochloride (100 mmoles·L^−1^), placed in a special well in the metabolic cage. Glycogen (liver and muscle) was extracted and hydrolysed with 30% KOH and saturated Na_2_SO_4_. The precipitated glycogen was re-suspended in distilled water, and incubated with 5% phenol and concentrated H_2_SO_4_ at room temperature, and the absorbance was measured at 490 nm^47^. The pattern of incorporated (^14^C)-glucose derived-(^14^C)-glycerol into total lipids from liver and epididymal adipose tissue was also evaluated. For this, total lipids were extracted from the tissue pellets with chloroform, and the neutral lipids and phospholipids were separated on silica gel thin-layer plates (TLC). The TAG fraction was revealed by staining with iodine vapours and this lipid fraction was scraped into vials^48^.

### Calculations and statistics

Cytoplasmic and mitochondrial NAD/NADH ratios were calculated as follows: NAD/NADH = [oxidized substrate]/[reduced substrate]·1/Keq, taking into account equilibrium constants for lactate and β-hydroxybutyrate dehydrogenases^49^. Linear regression analyses were made using the equation from Pearson’s approach. Results are expressed as mean ± SD, and statistical significance of the differences was assessed by two-way ANOVA for a normal distribution of data. Then, a Newman Keuls test was further applied and a *p* < 0.01 value was considered as significant.

## Supplementary Information

Supplementary Information.

## Data Availability

All data generated or analysed during this study are included in a Supplementary Information File.
